# Scutellarin ameliorates diabetic nephropathy via TGF-β1 signaling pathway

**DOI:** 10.1007/s13659-024-00446-y

**Published:** 2024-04-24

**Authors:** Bangrui Huang, Rui Han, Hong Tan, Wenzhuo Zhu, Yang Li, Fakun Jiang, Chun Xie, Zundan Ren, Rou Shi

**Affiliations:** 1 Department of Endocrinology, The First Affiliated Hospital of Kunming Medical University, Kunming, 650032 People’s Republic of China; 2 Key Laboratory of Medicinal Chemistry for Natural Resource (Ministry of Education)Yunnan Provincial Center for Research and Development of Natural Products, School of Pharmacy, Yunnan University, Kunming, 650500 People’s Republic of China

**Keywords:** Scutellarin, Diabetic nephropathy, Proteinuria, Fibrosis, Podocyte injury

## Abstract

Breviscapine, a natural flavonoid mixture derived from the traditional Chinese herb *Erigeron breviscapus* (Vant.) Hand-Mazz, has demonstrated a promising potential in improving diabetic nephropathy (DN). However, the specific active constituent(s) responsible for its therapeutic effects and the underlying pharmacological mechanisms remain unclear. In this study, we aimed to investigate the impact of scutellarin, a constituent of breviscapine, on streptozotocin-induced diabetic nephropathy and elucidate its pharmacological mechanism(s). Our findings demonstrate that scutellarin effectively ameliorates various features of DN in vivo, including proteinuria, glomerular expansion, mesangial matrix accumulation, renal fibrosis, and podocyte injury. Mechanistically, scutellarin appears to exert its beneficial effects through modulation of the transforming growth factor-β1 (TGF-β1) signaling pathway, as well as its interaction with the extracellular signal-regulated kinase (Erk) and Wnt/β-catenin pathways.

**Graphical Abstract:**

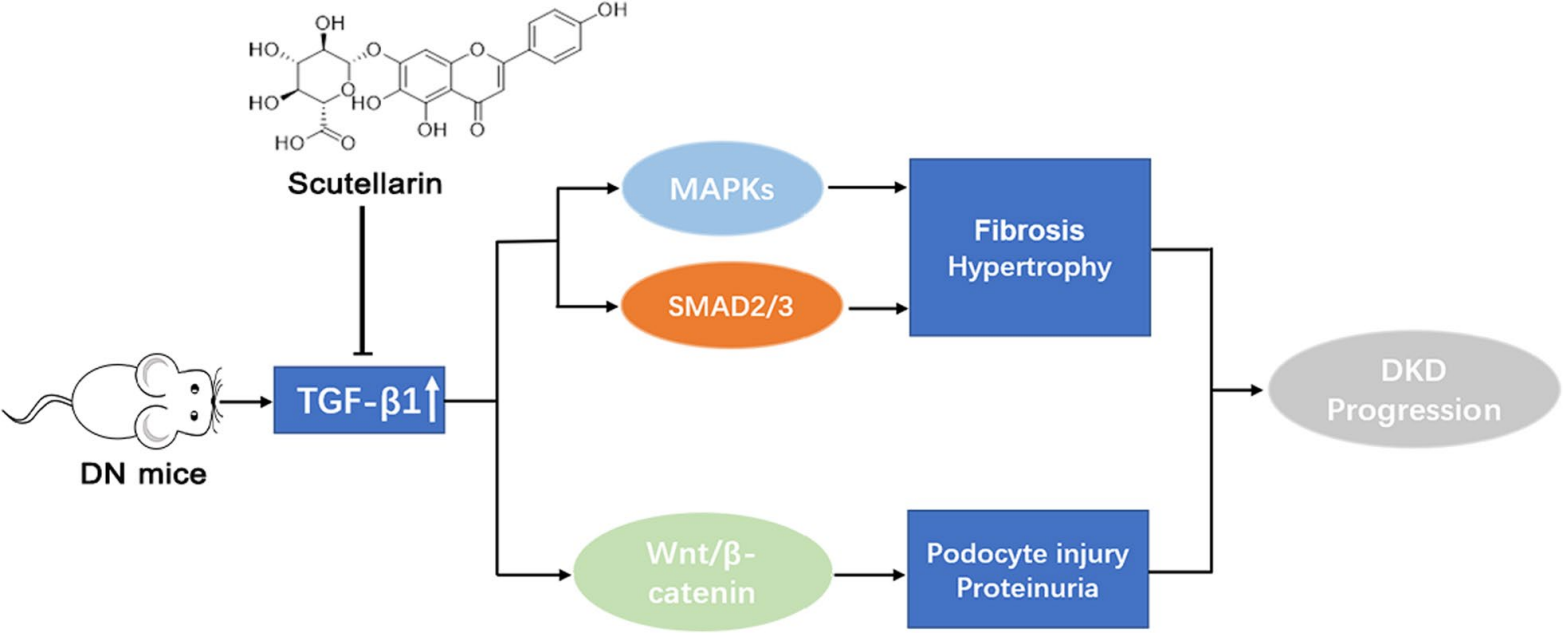

## Introduction

Diabetic nephropathy (DN) represents a significant microvascular complication of diabetes mellitus and stands as a prominent cause of terminal renal disease worldwide. This condition is associated with increased mortality and morbidity rates [1–3].

DN is characterized by a variety of pathological changes. These include glomerulosclerosis, mesangial dilatation, thickening of the glomerular basement membrane, tubulointerstitial fibrosis, podocyte damage and extracellular matrix accumulation [4, 5]. Notably, the development of progressive proteinuria is a prominent feature of DN, and effectively preventing and managing proteinuria presents a significant challenge in DN treatment [6]. The American Society of Nephrology (ASPN) Clinical Practice Guidelines recommend using the urine albumin-to-creatinine ratio as a screening tool for kidney disease in adults with diabetes mellitus [7].

Mesangial dilatation and renal fibrosis are key pathological features in the progression of DN [8]. Mesangial dilatation plays a crucial role in causing DN, characterized by aberrant proliferation of mesangial cells and accumulation of matrix proteins. This process is associated with the expression of alpha smooth muscle actin (α-SMA) in activated mesangial cells [9, 10]. Furthermore, the accumulation of matrix proteins like collagen III and fibronectin, within the mesangium contributes to the development of DN [11, 12]. In addition to mesangial dilatation, chronic kidney disease, including DN, often involves fibrotic changes in the glomerulus and tubulointerstitium [13, 14]. α-SMA, fibronectin, and collagen III are also implicated in renal fibrosis.

A central player in the progression of kidney fibrosis, a characteristic feature of chronic kidney disease including DN, is the transforming growth factor-β (TGF-β) signaling pathway [15]. Elevated expression of profibrotic TGF-β1 was observed in progressive forms of human kidney disease, highlighting its significance in fibrosis progression [16, 17]. Inhibiting TGF-β1 or its downstream signaling pathways has been shown to restrict renal fibrosis, while overexpression of TGF-β1 leads to fibrotic changes. The canonical downstream molecules of TGF-β1 signaling, SMAD2, SMAD3, and SMAD4 [18, 19], formation of a complex that translocates to the nucleus for regulation of downstream proteins [18, 20]. Additionally, TGF-β/Smad signaling crosstalks with other signaling pathways, including the Wnt/β-catenin signaling, the mitogen-activated protein kinase (MAPK) signaling and the mammalian target of rapamycin (mTOR) signaling. The complex pathogenesis of diabetic nephropathy still lacks a promising targeted treatment, despite advances in understanding the contribution of TGF-β1 and its downstream effectors.

Breviscapine, a prescription drug as a mixture of the natural flavonoid (contains ≥ 90% scutellarin and ≤ 10% apigenin-7-*O*-glucronide) derived from the traditional Chinese herb Erigeron breviscapus [21], has gained attention for its pharmacological properties and potential therapeutic effects in various diseases, including diabetic nephropathy. Breviscapine has exhibited a wide range of pharmacological activities, particularly in cardiovascular and hypertensive diseases. In the context of DN, breviscapine has been shown to have renoprotective effects by reducing the levels of urinary protein, blood urea nitrogen and serum creatinine, as well as regulating dyslipidemia parameters such as cholesterol, triglycerides, and high-density lipoproteins [22–24]. The combined medications of breviscapine with valsartan showed some enhanced protection against DN [25]. And it has been demonstrated another function in reducing urinary micro-albuminuria and improve renal function in DN. Other Studies demonstrated that breviscapine alone or combined with the angiotensin converting enzyme(ACE) inhibitor, nalapril, ameliorated streptozotocin-induced DN [26]. These effects suggest that the injection of breviscapine benefit to patients with DN. In addition, Scutellarin has been reported to protect against kidney injury [27–30] via its anti-inflammation and antioxidant activity.

However, the active component responsible for its anti-DN properties remains uncertain, and the underlying regulatory mechanisms remain unclear and even confusing. Scutellarin, a prominent constituent of breviscapine, has been identified as a potential candidate for mediating the therapeutic effects. Future research directions and the need for in-depth studies on scutellarin as the active component of breviscapine are emphasized to unravel the complex mechanisms underlying the therapeutic effects of breviscapine in DN.

## Methods and materials

### Materials and reagent

Scutellarin (Yunnan Phytopharmaceutical Co., LTD., China); Empagliflozin (Cat. C14295412, Macklin Biochemical, China), Streptozotocin (Cat. S8050, Solarbio, China); goat anti-rabbit immunoglobulin G (IgG) horseradish peroxidase (HRP)-linked antibody (Cat. AS014, Abclonal, China); anti-mouse IgG HRP-linked antibody (Cat. 7076S, Cell Signaling Technology, USA); Methenamine Silver Plating Stain Kit (Cat. G1790, Solarbio); Glycogen Periodic Acid Schiff (PAS) Stain Kit (Cat. G1281, Solarbio); Masson’s Trichrome Stain Kit (Cat. G1340, Solarbio); Mouse MAU enzyme-linked immunosorbent assay (ELISA) Kit (Cat. JL20493, JONIN, China); Anti-DKK1 antibody (Cat. A00632, Boster Biological Technology, China); Anti-SNAIl antibody (Cat. BP0449, Boster Biological Technology); Anti-α-SMA antibody (Cat. BM0002, Boster Biological Technology); Anti-TGFB1 antibody (Cat. BA0290, Boster Biological Technology); Anti-NPHS1 antibody (Cat. BA1669, Boster Biological Technology); Anti-NPHS2 antibody (Cat. BA1688, Boster Biological Technology); Anti-SMAD2/3 antibody (Cat. BA1395, Boster Biological Technology); Anti-Phospho-SMAD2(s250) antibody (Cat. BM4693, Boster Biological Technology); Anti-FN1 antibody (Cat. BA1772, Boster Biological Technology); AXIN2 antibody (Cat. Ab32197, Abcam, USA); Anti-Phospho-SMAD3(ser425) antibody (Cat. AF3362, Affinity Biosciences, China); Anti-COL3A1 antibody (Cat. M00788, Boster Biological Technology); Anti-extracellular signal-regulated kinase (ERK)1/2 antibody (Cat. ET1601-29, HUABIO, China); Anti-ERK1(PT202/PY204) + ERK2(PT185/PY187) antibody (Cat. ET1610-13, HUABIO); Anti-P38 antibody (Cat. ET1602-26, HUABIO); Anti-Phospho-P38(Thr180 + Tyr182) antibody (Cat. ER1903-01, HUABIO); Anti-β-Actin antibody (Cat. EM21002, HUABIO); Anti-SMAD4 antibody (Cat. A5657, Abclonal); Anti-β-catenin antibody (Cat. 610154, BD Biosciences, China); Creatinine (Cr) Assay kit (Cat. C011-2-1, Nanjing Jiancheng Bioengineering Institute, China); Glucose Assay Kit (Cat. F006-1-1, Nanjing Jiancheng Bioengineering Institute); microalbunminuria (MAU) ELISA kit (Cat. JL20493, JONIN).

### Induction of DN mice

C57BL/6J male mice of approximately 6 weeks of age were obtained from the Laboratory Animal Center of Yunnan University (Kunming, China) and housed at 23 °C with 50% humidity and a 12 h light/12 h dark cycle; The experimental procedures were approved by the Institutional Animal Care and Use Committee, Yunnan University (Protocal: YNU20220267). After adaptation, 8-week-old mice were induced with 60 mg/kg streptozotocin (STZ) diluted in citrate buffer (0.1 mol/L, pH 4.5) by intraperitoneal injection for 3 consecutive days, while the same volume of citrate buffer was given to the control mice. the urinary albumin level and blood glucose level were measured at 18-week-old mice, all animals were randomized into five groups (n = 9–10 per group): STZ group, control group, STZ + Empagliflozin (Empa) group (20 mg/kg/day), and STZ + Scutellarin (Scu) groups (10 or 40 mg/kg/day). Mice were given scutellarin and empagliflozin daily by gavage from week 18 to week 24 in a 1:9 solution of dimethyl sulfoxide (DMSO): water. The same volume of vehicle (DMSO:water = 1:9) was administered by gavage to mice in both the control and STZ model groups.

### Histopathology and immunohistochemistry

After the experiments, kidney samples were rapidly excised, followed by separation of renal cortices. Renal histological lesions were then obtained from the right renal cortex. Briefly, kidneys were fixed in 4% paraformaldehyde and paraffin-embedded. The sections were subsequently cut at 5 μm and stained with periodic acid silver methenamine (PASM) stain, Masson’s trichrome (MS) stain and periodic acid-Schiff (PAS) stain.

Glomerular areas were assessed in samples by masked PAS staining and mesangial index quantified using Image-Pro Plus 6.0 software (mesangial area/total glomerulus area × 100).

For immunohistochemistry, kidney tissue slides were deparaffinized and rehydrated, exposed to Ethylenediaminetetraacetic acid (EDTA) antigen retrieval solution for 20 min (95–100 °C), washed once with phosphate buffered saline (PBS), treated with 3% H_2_O_2_ for 15 min, washed with PBS, and then treated with 5% bovine serum albumin for 30 min at 37 °C, and incubated with anti-FN1, anti-α-SMA, anti-NPHS1 or anti-NPHS2 overnight at 4 °C. The samples were visualized by diaminobenzidine staining after washing and incubation with secondary antibodies.

### Measurement of blood glucose

After fasting 4–6 h, serum samples of mice from tail vein were collected. Then the samples were measured for blood glucose using Glucose Assay Kit.

### Quantification of MAU

Urine samples were collected for 24 h and urinary albumin excretion was measured using a mouse MAU ELISA kit according to the manufacturer's instructions.

### Western blotting

Kidney tissues were homogenized in lysis buffer and processed for western blotting. The membranes were incubated with primary antibodies to NPHS1, NPHS2, p38, phosphor-p38, α-SMA, SMAD4, snail1, ERK1/2, phospho-ERK1/2 or β-actin overnight at 4 °C. This was followed by hybridization with secondary antibodies for 2 h at room temperature. Bands were visualized using ECL and quantified using ImageJ software.

### Statistical analysis

All data are expressed as mean ± standard deviation (S.D.). This study used pairwise comparisons by Tukey’s test method. GraphPad Prism 8.0 software was used for statistical analysis.

## Results

### Scutellarin ameliorates proteinuria, glomerular expansion and mesangial matrix of DN mice

**Fig. 1:**
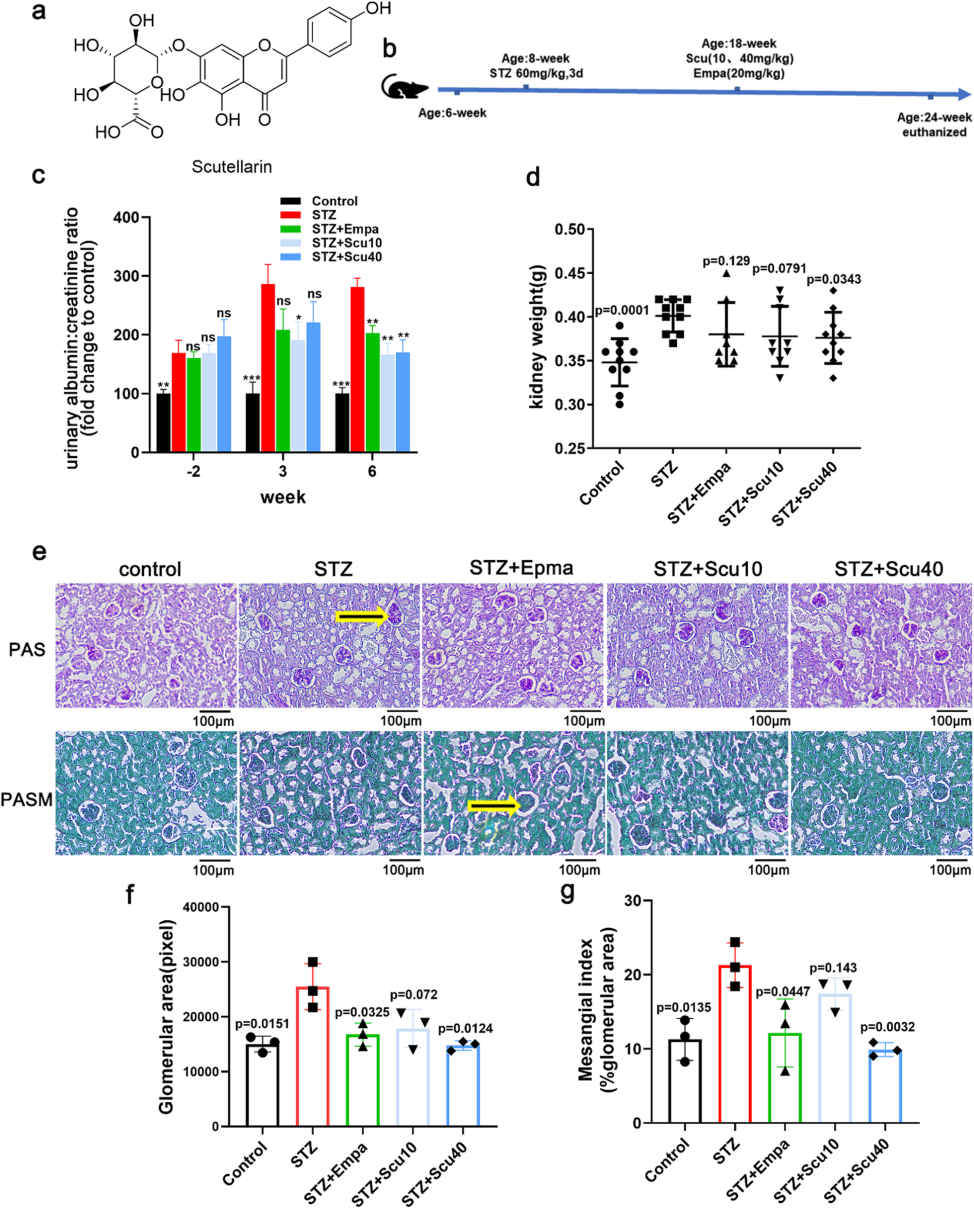
Scutellarin Ameliorated Proteinuria, Glomerular Expansion and Mesangial Matrix of the DN Mice. **a** The chemical structure of Scutellarin. **b** Work-flow of the experiment. **c** Ratio of urinary albumin: creatinine of the mice treated with vehicle (STZ), low dose of scutellarin (10 mg/kg, STZ + Scu10), high dose of scutellarin (40 mg/kg, STZ + Scu40) or empagliflozin (20 mg/kg, positive control, STZ + Empa). The samples were collected at the before, the 3rd week and the 6th week of the treatments. **d** Kidney weights of the mice. **e** Representative PAS or PASM-stained images of the kidney samples from the mice as indicated (× 200, scale bar = 100 µm). **f**, **g** Quantifications of the glomerular area and mesangial matrix of the samples from D. Data were summarized as mean ± S.D.; n = 3–10 for each group, “n” stands for the number of animals; *p < 0.05, **p < 0.01, ***p < 0.001 vs. the model group (STZ); ns, non-significant, p vs. the model group (STZ)

DN mice were established by 60 mg/kg of STZ once a day for 3 days and the serum glucose levels of the 8-week mice were above 15 mmol/L. Following by 10-week’s feeding and the urinary albumin/creatinine ratios were significantly higher than the mice treated with vehicle only. Then, these mice were treated with low (10 mg/kg), high (40 mg/kg) doses of scutellarin, empagliflozin (20 mg/kg, positive control) or vehicle for another 6 weeks. At the 3rd week of the 6-week’s treatments with the drugs, the urinary albumin/creatinine ratios were measured once. After the treatments for 6 weeks, the mice were euthanized and the urine samples, kidneys were collected for evaluation of the effects of the drugs on the DN mice at the end stage of the treatments (Fig. 1b).

As showing, the ratios of urinary albumin/creatinine of the model mice of DN were maintaining high during the drug treatments. Interestingly, the urinary albumin/creatinine ratios of the mice treated with the both doses of scutellarin were tended to be ameliorated at the 3rd week, and the low, high dose of scutellarin or empagliflozin-treated mice showed significant decreases of urinary albumin/creatinine after 6-week’s treatments (Fig. 1c). Besides, the kidney weights of the mice were decreased to the certain degrees (Fig. 1d), even though the kidney weight is not as sensitive as the ratio of urinary albumin/creatinine for the features of DN.

In general, STZ-induced DN mice show a feature of developed glomerular hypertrophy in kidneys. Here, revealed by the PAS and PASM stainings, we also observed the enlarged glomerular area and the expanded mesangial matrix of the DN mice (Fig. 1e–g). Under the conditions, low dose of scutellarin tended to induce decrease of glomerular expansion and mesangial matrix, whereas the treatments with a high dose of scutellarin significantly improved these two pathological features of DN, similar to the empagliflozin-treated DN mice (Fig. 1e–g).

### Scutellarin ameliorates renal fibrosis of the DN mice

**Fig. 2:**
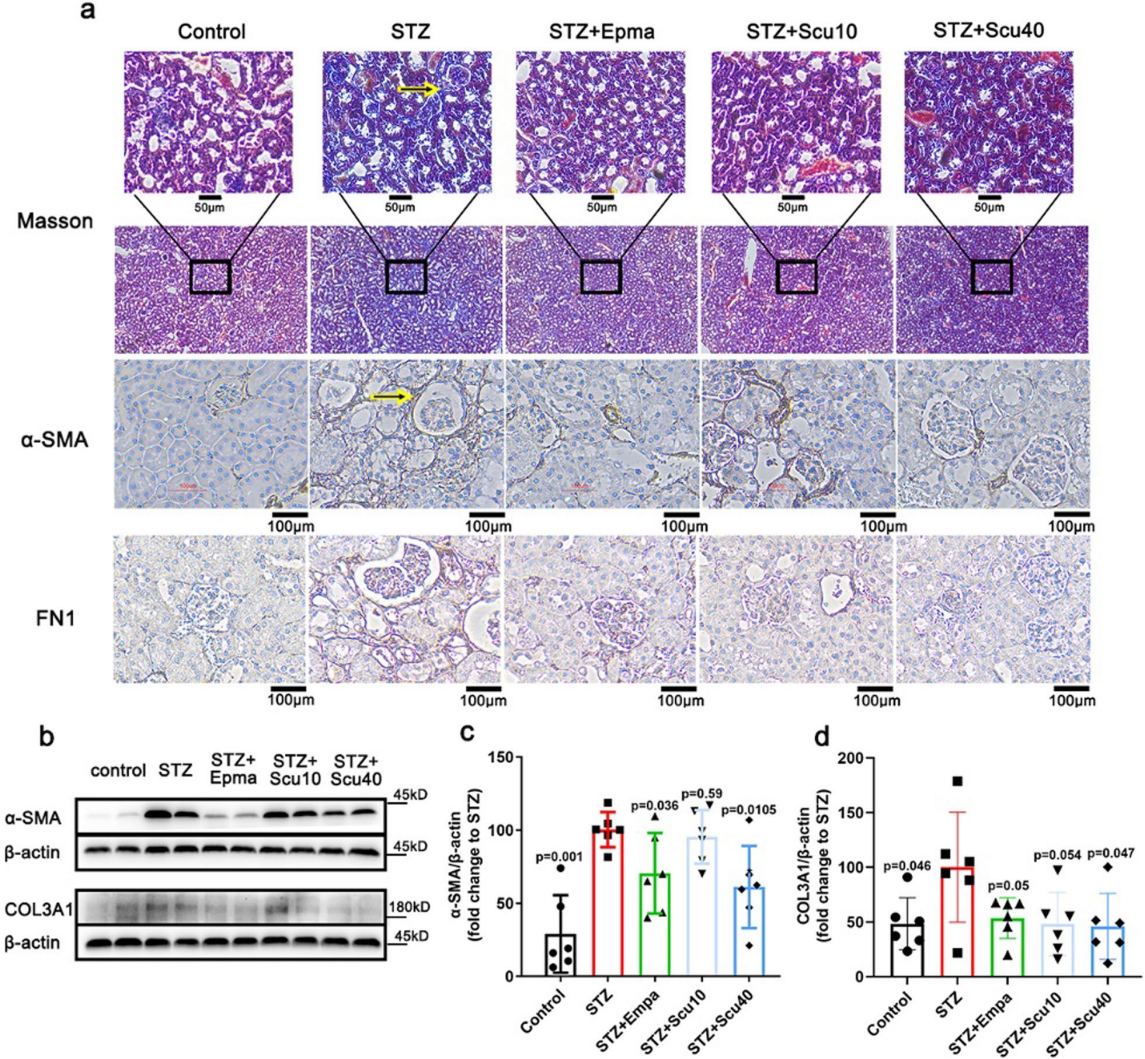
Scutellarin Improved Renal Fibrosis of the DN Mice. **a** Masson’s trichrome staining (× 200; scale bar = 50 µm) and immunohistochemistry staining for α-SMA and FN1 (× 200; scale bar = 100 µm) of the mice treated with vehicle, scutellarin or empagliflozin. **b** Representative western-blot images for α-SMA and Col3A1 of the mice. **c**, **d** Quantifications of the protein levels of α-SMA and Col3A1, respectively. β-Actin used as a loading control. All data are normalized to the STZ group and presented as the mean ± S.D.; n = 6 for each group, “n” stands for the number of animals; p vs. the model group (STZ)

Fibrosis is a typical feature of DN. To estimate the effect of scutellarin on fibrosis, we measured fibrotic markers Col3A1, α-SMA and FN1. As showing by Masson staining, fibrosis was observed in DN mice, whereas scutellarin ameliorated the histopathological feature of the fibrosis (Fig. 2a). Besides, revealed by immunohistochemistry stainings, two typical markers of fibrosis, α-SMA and FN1, were remarkably suppressed (Fig. 2a). To further confirm the downregulations of the markers, we performed western-blotting for measuring the proteins α-SMA and Col3A1. As expected, while α-SMA and Col3A1 were upregulated in the STZ-induced DN mice, low dose of scutellarin tended to downregulate the two markers (Fig. 2b–d). Furthermore, high dose of scutellarin significantly induced the downregulations of the proteins, similar to that of positive control empagliflozin, (Fig. 2b–d).

**Fig. 3:**
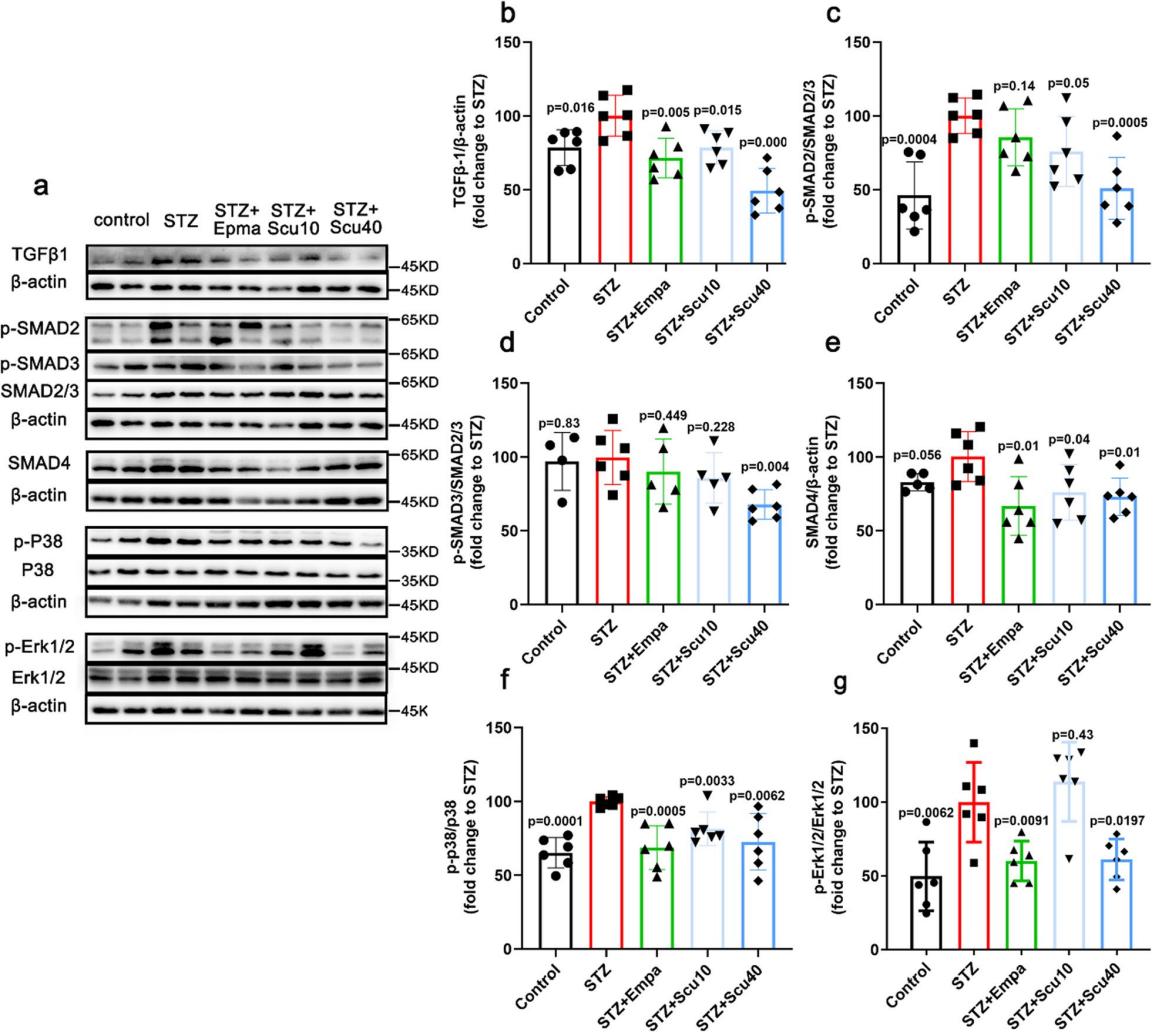
Scutellarin Inhibits TGF-β1 and Its Downstream Signalling Pathway. **a** Representative images of Western blotting samples for TGF-β1, p-SMAD2, p-SMAD3, SMAD2/3, SMAD4, p-p38, p38 and p-Erk and Erk1/2 of the mice treated with vehicle, scutellarin or empagliflozin. **b**–**g** Quantifications of the protein as indicated. All data are normalized to the STZ group and presented as the mean ± S.D.; n = 4–6 for each group, “n” stands for the number of animals; p vs. the model group (STZ)

Since scutellarin remarkably ameliorated renal fibrosis in STZ-induced DN mice. We further elucidated the underlying mechanism of the effect. It has been reported that TGF-β1 signaling is involved in the effect of breviscapine on DN [26]. Here we also screened the key signaling proteins of the TGF-β1 pathway, including TGF-β1 and its downstreams, SMAD2/3, p-SMAD2, p-SMAD3, SMAD4, Erk1/2 and p-Erk1/2, p38 and p-p38. As expected, the TGF-β1 was upregulated in the model group, whereas both low and high doses of scutellarin caused the downregulations of the protein, similar to that of empagliflozin treatments (Fig. 3a, b). Furthermore, p-SMAD2 increased by DN, and the increase was suppressed by the high dose of scutellarin but not the low dose of scutellarin and empagliflozin (Fig. 3a, c). Although the change in p-SMAD3 was not observed in the low dose of scutellarin and empagliflozin, but the high dose of scutellarin significantly reduced p-SMAD3(Fig. 3a, d). Meanwhile, the proteins levels of SMAD4 (Fig. 3e), p-p38 (Fig. 3f) and p-Erk (Fig. 3g) were shown similar tendency as that of TGF-β1, except that the low dose of scutellarin did not induce a significant downregulation of p-Erk (Fig. 3g).

### Scutellarin improved podocytes injure in DN mice

**Fig. 4:**
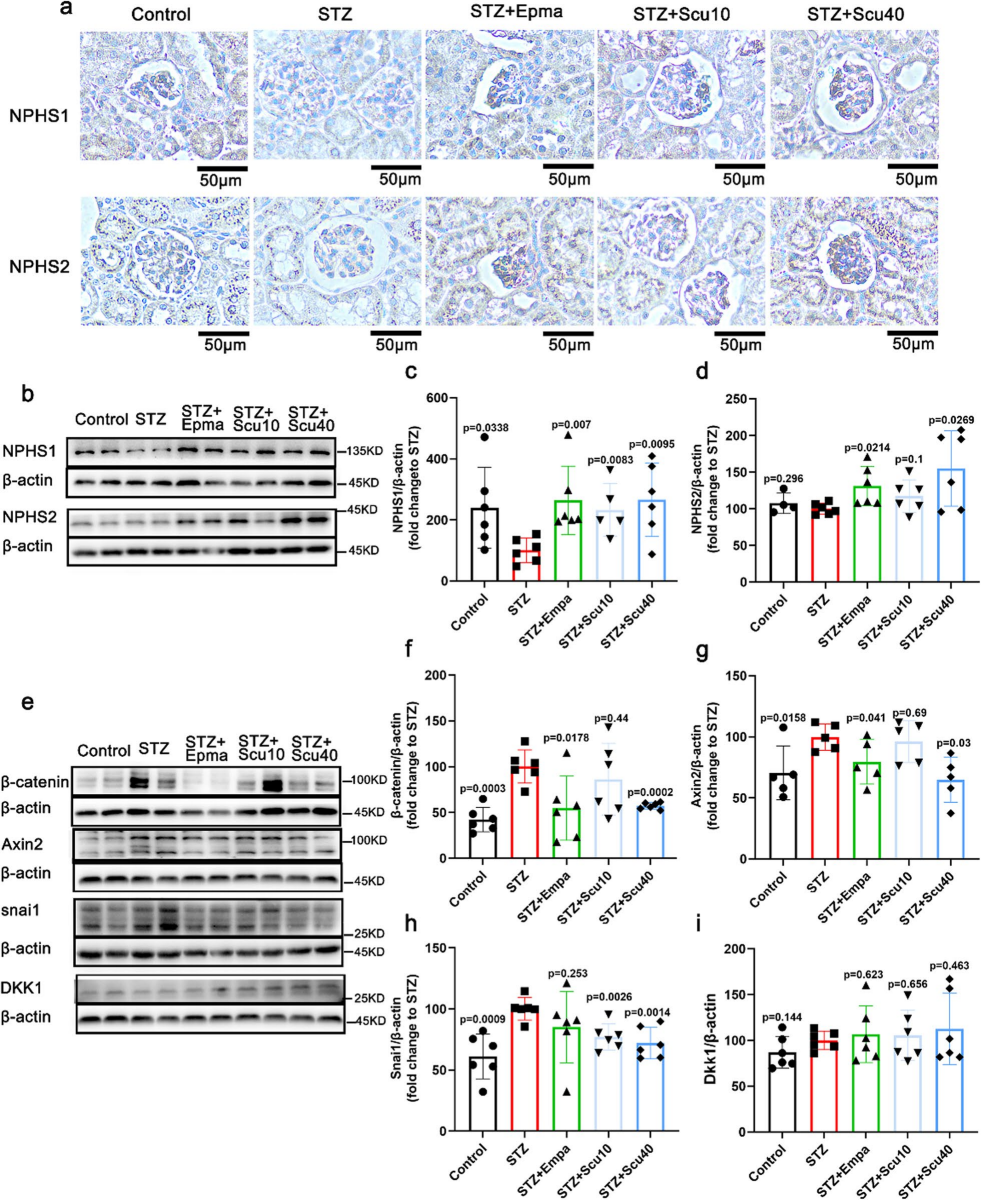
Scutellarin Restored Podocyte Injury of the DN Mice. **a** Representative images of immunohistochemistry for NPHS1 and NPHS2 of the mice treated with vehicle, scutellarin or empagliflozin (× 200; scale bar = 50 µm). **b** Representative images of Western-blotting for NPHS1, NPHS2. **c** Quantitative plot of the expression of NPHS1 of the mice. **d** Quantitatification of NPHS1 expression of the mice. **e** Representative images of Western-blotting for β-catenin, Axin2, snail and DKK1 of the mice. **f**–**i** Quantifications of the protein levels for β-catenin, Axin2, snail and DKK1 from E. All data are presented as the mean ± S.D.; n = 4–6 for each group, “n” stands for the number of animals; p vs. the model group (STZ)

NPHS1 and NPHS2 are two key markers for podocyte injury or activation, in order assess the impact of scutellarin on podocyte injury in the DN mice, we used immunohistochemical staining to determine the protein levels of NPHS1 and NPHS2 in the kidneys of the mice, and found that NPHS1 was downregulated in the model mice, whereas the treatments with scutellarin or empagliflozin significantly restored the expressions of NPHS1 back to the control level (Fig. 4a). In consistent with the immunohistochemistry result, the western-blotting of the protein demonstrated that the treatments with scutellarin or empagliflozin significantly restored the down regulated NPHS1 in STZ-induced DN mice (Fig. 4b, c). For NPHS2, the immunohistochemistry result was similar to the Western blot result, showing that treatment with empagliflozin or high dose scutellarin induced significantly higher expression of NPHS2 (Fig. 4b, d), but the level of NPHS2 was not changed in DN mice.

Numerous studies have demonstrated that Wnt/β-catenin signaling mediates TGF-β1-driven podocyte injury and proteinuria [31, 32]. Axin, β-catenin, Dkk, and Snail, are the key proteins of the Wnt/β-catenin signaling pathway. Here we continued to detect Wnt/β-catenin signaling in podocytes of the mice. As shown, the western-blotting data demonstrated that β-catenin was upregulated in the DN mice, whereas high dose of scutellarin or positive control empagliflozin decreased its expressions (Fig. 4e, f). These changes of β-catenin were similar to that of Axin2 (Fig. 4e, g). The difference is that empagliflozin-treated mice showing only a trend of decrease for Snail (Fig. 4e, h), but the increase in Snail was suppressed by the low and high doses of scutellarin. However, DKK1 was not changed in DN mice, so that no changes were observed for the treatments with vehicle, scutellarin or empagliflozin (Fig. 4e, i).

## Discussion

In this study, we have provided a comprehensive and systematic analysis demonstrating the protective effects of scutellarin against various aspects of DN in STZ-induced DN mice. Our findings indicate that scutellarin effectively mitigates proteinuria, renal hypertrophy, fibrosis, and podocyte injury. Furthermore, the pharmacological data strongly supported the participation of the TGF-β1 signaling pathway and its downstream network in mediating the observed effects of scutellarin. These results highlight scutellarin as the key component of breviscapine responsible for regulating DN.

Previous reports have shown that breviscapine's beneficial effects in treating DN. Breviscapine treatment has been shown to effectively inhibit the progression of tubulointerstitial injury, albuminuria and glomerular hypertrophy. Western blot analysis showed a significant reduction in the expression of TGF-β1 [33]. Additionally, it has been reported that combining breviscapine with enalapril resulted in superior renoprotective effects compared to individual treatments in rats with DN. The mechanism underlying this synergistic effect may involve the suppression of increased oxidative stress, PKC activity, and the overexpression of TGF-β1 in renal tissue [26].

The role of TGF-β1 in DN pathogenesis is well established. TGF-β1 plays a crucial role in the production of extracellular matrix in the kidney and its dysregulation has been linked to the progression of renal fibrosis in DN [34]. Mice with overexpression of TGF-β1 specifically in renal tubular epithelial cells develop spontaneous systemic tubulointerstitial fibrosis, demonstrating that TGF-β1 contributes to renal fibrotic processes [35]. Our results showed that scutellarin markedly suppressed the overexpression of TGF-β1 and its downstream molecules, suggesting its potential to improve renal fibrosis in diabetic nephropathy.

Podocytes contribute to the development of albuminuria in DN through their critical role in maintaining the integrity of the glomerular filtration barrier [36]. Reduced expression of nephrin (NPHS1) and podocin (NPHS2) is associated with fusion of the podocyte foot processes, this leads to breakdown of the glomerular filtration barrier and subsequent albuminuria [37]. We found that scutellarin treatment significantly restored the expressions of NPHS1 and NPHS2 in the glomerulus. Wnt/β-catenin signaling involved in TGF-β1-induced proteinuria and podocyte injury, according to new evidence [31, 32]. Several key proteins, including Axin, β-catenin, Dkk, and Snail, are integral components of the Wnt/β-catenin signaling pathway. β-Catenin is a cytoplasmic protein that translocates to the nucleus, mediating TCF-LEF-dependent gene expression [38]. Snail is one of the target genes regulated by the Wnt/β-catenin signaling pathway and has been implicated in kidney injury [38]. Axin is part of the destruction complex' responsible for degrading β-catenin when Wnt ligands fail to act [38]. On the other hand, Dkk acts to inhibit Wnt/β-catenin signalling [38]. In this study, we noticed that scutellarin treatment markedly reduced the protein expressions of β-catenin, Axin2, and Snail in the glomerulus. However, the protein expression of Dkk1 was not notably altered by scutellarin treatment. These findings suggest that scutellarin may regulate Wnt/β-catenin signaling in podocytes, possibly by suppressing the activation of β-catenin and its downstream target genes, including Axin2 and Snail.

We set up two different doses of scutellarin to investigate its effects on DN. A high dose group (STZ + Scu40) and a low dose group (STZ + Scu10) were established to assess the dose-dependent response of scutellarin. The results indicated that the high dose group exhibited significant improvements in pathological indicators and key proteins involved in the signaling pathway compared to the low dose group. For instance, the low dose of scutellarin significantly reduced the urinary albumin/creatinine ratio, suggesting a beneficial effect on albuminuria. However, it only showed a tendency to decrease glomerular expansion and mesangial matrix, without reaching statistical significance. Similarly, the low dose of scutellarin significantly reduced the expression of TGF-β1, which is involved in the pathogenesis of DN, but it did not significantly affect the proteins in its downstream signaling pathway. There could be several possible reasons for these observations. Firstly, it is likely that the low dose of scutellarin had weaker efficacy in mitigating the pathological phenotypes compared to the high dose. Secondly, the effects of scutellarin on molecular-level proteins may be less sensitive at the lower dose, resulting in a lack of significant changes in the downstream signaling pathway proteins.

Moreover, several clinical guidelines recommend the use of the SGLT2 inhibitor empagliflozin for the treatment of DN. In our study, we compared the effects of empagliflozin with scutellarin at a high dose and found relatively comparable effects in alleviating the pathological features of DN. However, it is worth noting that the effect of empagliflozin on the pathway was not completely identical to that of scutellarin. Specifically, empagliflozin did not significantly affect the downstream signaling pathway of TGF-β1, which is a key pathway involved in the pathogenesis of DN. A study conducted by Issei Tomita and colleagues suggests that SGLT2 inhibitors mediate the protection of DN through the promotion of ketone body-induced mTORC1 inhibition [6].

In conclusion, our study provides compelling evidence for the promising preventive effect of scutellarin on DN. Scutellarin demonstrated its effectiveness by inhibiting the TGF-β1 profibrotic signaling pathway and suppressing the phosphorylation levels of MAPKs (ERK1/2 and p38) as well as the Wnt/β-Catenin pathway (β-Catenin, Axin2). These findings indicate that scutellarin acts on multiple molecular targets involved in the pathogenesis of DN. However, despite these significant findings, the specific protein targets of scutellarin in improving DN remain unclear and require further exploration. The detailed molecular mechanisms by which scutellarin exerts its renoprotective effects require further study.

## Data Availability

All relevant data are within the manuscript.
